# The direction of research into visual disability and quality of life in glaucoma

**DOI:** 10.1186/1471-2415-11-19

**Published:** 2011-08-04

**Authors:** Fiona C Glen, David P Crabb, David F Garway-Heath

**Affiliations:** 1 Department of Optometry and Visual Science, City University London, UK; 2 NIHR Biomedical Research Centre for Ophthalmology, Moorfields Eye Hospital NHS Foundation Trust and UCL Institute of Ophthalmology, London, UK

## Abstract

**Background:**

Glaucoma will undoubtedly impact on a person's ability to function as they go about their day-to-day life. The purpose of this study is to investigate the amount of published knowledge in quality of life (QoL) and visual disability studies for glaucoma, and make comparisons with similar research in other chronic conditions.

**Methods:**

A systematic literature search of the Global Health, EMBASE Psychiatry and MEDLINE databases. Title searches for glaucoma and six other example chronic diseases were entered alongside a selection of keywords chosen to capture studies focusing on QoL and everyday task ability. These results were further filtered during a manual search of resulting abstracts. Outcomes were the number of publications per year for each disease, number relating to QoL and type of glaucoma QoL research.

**Results:**

Fifteen years ago there were no published studies relating to the impact of glaucoma on QoL but by 2009 this had risen to 1.2% of all glaucoma articles. The number of papers relating to QoL as a proportion of all papers in glaucoma in the past 10 years (0.6%) is smaller than for AMD and some other disabling chronic diseases. Most QoL studies in glaucoma (82%) involve questionnaires.

**Conclusion:**

QoL studies in glaucoma are increasing in number but represent a tiny minority of the total publications in glaucoma research. There are fewer QoL articles in glaucoma compared to some other disabling chronic conditions. The majority of QoL articles in glaucoma research use questionnaires; performance-based measures of visual disability may offer an additional method of determining how the disease impacts on QoL.

## Why study quality of life and disability in glaucoma?

Definitions of visual impairment centre largely on the application of numerical values to classify disease state and pay less attention to what this means in terms of the individual and the types of disability they may face as a result of visual loss. For instance, whilst clinical measures of visual function such as visual fields facilitate the monitoring of disease severity in glaucoma, they offer less insight into the perceived or actual impact of the disease on the individual as they go about everyday tasks. Yet, this information will be highly valued by the patient: *How will the disease impact on their quality of life? How will their visual loss affect them psychologically? Will they still be able to read or drive? Will they be able to recognise their loved ones? *The reality appears to be that decreased visual functioning can have a wide number of disabling consequences on an individual's daily life, including increased likelihood of injury from falls or automobile accidents [1-3] and a decreased ability to carry out activities of daily living [4-7], which can in turn lead to reduced confidence in or even avoidance of activities the patient once deemed important and pleasurable [8,9]. Since quality of life (QoL) - the sense of personal satisfaction with the conditions in which one lives - is likely intertwined with how well the individual is able to carry out the activities of daily living that are most important to them, the need for a better understanding into the types of visual disability faced by individuals with glaucoma is glaring.

Establishing how the disease is likely to affect QoL is not simply a task of using clinical measures of visual function alone. For example, each patient is likely to present a different pattern of impairment (visual disability) depending on the combination of individual characteristics of the disorder (such as contrast sensitivity, visual field defect severity, colour vision deficits) that they present. Likewise, general health, socio-economic factors, personality characteristics, coping strategies, the value placed on particular tasks and other factors will also influence how the disease will impact on that individual's daily life [10,11]. Thus, only by direct investigation into perceived and measured problems experienced by visually impaired individuals can informed insight be gained into the types of disability experienced in everyday life. This information is invaluable, since it offers a means for the clinician to inform newly diagnosed individuals with regards to future changes to their lives and possible strategies that they could employ to maximise their independence and increase their QoL.

Glaucoma is a leading cause of irreversible vision impairment with the number affected expected to increase substantially in the future due to the aging of the population [12]. Insight into how glaucoma affects QoL might offer a means of developing treatment strategies tailored towards the individual's needs. QoL also has health economic implications; more precise knowledge of the impact on QoL will help, for example, determine the level of disease at which the benefit of screening for the disease outweighs costs [13]. It has also been shown that good vision is valued much more than many physicians realise [14] and that ophthalmologists often underestimate the impact of the disorder on the patient's QoL [15]. The development of methods for investigating the functional ability and QoL of individuals with glaucoma is therefore vital for a better understanding of the condition for the ophthalmologist, the patient and for an economic rationale for how to deal with the condition. Research of this type is starting to gather momentum in the literature, and has been recently reviewed [16,17].

This study has three aims related to the published research activity that has examined the impact of glaucoma on disability and QoL to date. First, the study aims to examine the hypothesis that publications, as a surrogate of research activity in this area, are increasing in number. Second, the study seeks to investigate the hypothesis that this level of research activity in this area is equivalent to some other example chronic conditions including age-related macular degeneration (AMD). Third, the study considers the hypothesis that the methods typically used in this type of research are reliant on questionnaires and self report, leading to discussion on the alternative approach of performance-based measures which more directly assess patient functioning.

## Literature search strategy

A systematic literature search was conducted of the MEDLINE, EMBASE Psychiatry and Global Health databases using the Ovid search platform on the 16th March 2010 and included publications indexed up until the end of December 2009. Title searches were conducted for seven example chronic conditions (glaucoma, AMD, Type 2 Diabetes, rheumatoid arthritis, hearing impairment, Parkinson's Disease, Multiple Sclerosis) using the key words displayed in Table 1 over a ten year period (1999-2009). The search was extended to 20 years (1989-2009) for the purpose of examining the trend relating to glaucoma-related publications as a surrogate measure of research activity trends in this area. The results for each search were combined with the results from an abstract search using the following 'quality of life' key words or phrases: "quality of life", "functional consequences", "performance", "real world", "functional ability", "everyday", "daily living", "daily life", "behaviour", "behavior", "activities of daily living", "independent living". Results were restricted to those papers which contained one of the disease key words *and *at least one of the QoL keywords. There were no language or country restrictions and results were limited to a human population. Review articles and reference sections of key papers were carefully read to look for any other relevant papers which may not have been indexed in these databases.

**Table 1:** Disease key words

Search Number	Disease Key Words
1	"glaucoma" (1)
2	"age-related macular degeneration" *or *"AMD" *or *"age related macular disease" *or *"age related maculopathy" *or *"ARMD" *or *"macular degeneration" *or *"macular disease"
3	"type 2 diabetes" *or *"type II diabetes" *or *"type two diabetes" *or "*diabetes mellitus"
4	"hearing impairment" *or *"hearing loss" *or *"loss of hearing" *or *"deafness"
5	"rheumatoid arthritis" *or *"rheumatic gout" *or *"rheumatoid disease"
6	"parkinson's disease" *or *"parkinson disease"
7	"Multiple Sclerosis" *or *"MS" *or *"Acute Fulminating Sclerosis" *or *"Disseminated Sclerosis"

(1) Since synonyms for the disease all tend to contain the word 'glaucoma' (i.e. primary open angle *glaucoma*, normal-tension *glaucoma*, secondary *glaucoma*), it was assumed that the term "glaucoma" alone would be sufficient to encapsulate all forms of the disease

Keywords and phrases were chosen in an attempt to include as many relevant papers as possible, but their possible ambiguity and constraints relating to the search platform meant that a large number of irrelevant papers would also need to be excluded. A careful manual search of the abstracts resulting from the search was subsequently conducted. In order to be included in the final results the paper had to be a systematic research study with a strong emphasis on the *impact *of the disease on patient's daily life (review articles were not included). Papers where QoL was only used as a secondary measure or articles regarding 'visual function' as an outcome of clinical measures were excluded. Another exclusion criterion applied when the search terms yielded papers focusing on the monetary costs of the disease on, for example, the UK NHS or hospitals. Those studies assessing QoL as an outcome of a drug trial, surgery, rehabilitation method or other intervention were not included in these search results, because the number of QoL publications will be partially driven by the demand for medical interventions for a particular disorder. For instance, in order to judge the cost-effectiveness of the development of a new drug, a measurement of QoL is often needed to financially and ethically justify the intervention. Since finances will be allocated differently for different diseases, and demand for medical trials will vary, it was deemed inappropriate to include these types of studies for the sake of a comparison-based paper.

The total number of all papers published for each of the relevant years for each chronic disease (i.e. the number of papers each year with a disease keyword in the title) was also recorded. For example, this enabled the results of the glaucoma QOL studies to be considered as a percentage of the total number of 'glaucoma' themed articles (i.e. featuring the word 'glaucoma' in the title) for each year. Any difference in number of publications from year to year could thus most likely be attributed to interest in QoL instead of simply due to more studies in general being published every year. In addition, abstracts (and full texts if necessary) for resulting papers in the 20-year glaucoma search were examined for information regarding the type of methods used to investigate QoL in each study.

## General trend in QoL publications in glaucoma

**Figure 1:**
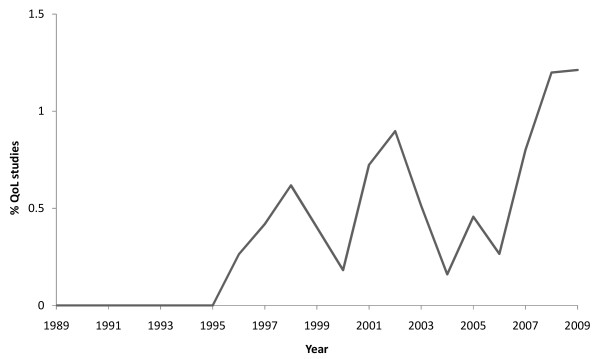
**Number of QoL papers per year as a percentage of all glaucoma articles**.

The initial literature search yielded a total of 294 papers for QoL and glaucoma. Following a careful manual search, 244 were excluded based on the additional filtering criteria leaving a total of 51 suitable papers. Figure 1 illustrates an increase in the number of QoL studies related to glaucoma between the early 1990s and the present year; 15 years ago no published studies existed on the subject, but since then a general increase has occurred with the highest percentage of glaucoma studies relating to QoL being published in 2009 (1.2%; n = 8 of 660 - total number of glaucoma studies that year). This obvious rise suggests that the relevance of studying QoL in glaucoma is beginning to be understood. However, it is remarkable to note that these studies examining the life of the patient beyond the clinic are a very small representation of the total number of glaucoma papers published each year; despite there being a higher proportion of glaucoma QoL themed papers in 2009 compared with earlier years, 1.2% is still undoubtedly tiny. Of course the importance of other key areas of research, such as the development of treatments and clinical diagnostic measures cannot be underestimated. However, this figure still seems anomalous for an area of research which encompasses the core of disease, which is how it actually manifests itself and impacts on an individual.

## QoL research in other chronic conditions

**Figure 2:**
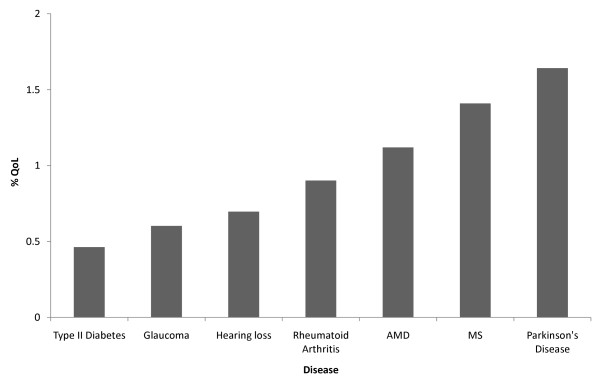
**Number of QoL studies as a percentage of all studies for 7 chronic diseases over last 10 years**.

To gauge if QoL type research in glaucoma was neglected more than in other chronic conditions, the number of publications over the last 10 years for AMD, hearing impairment, Parkinson's Disease (PD), Multiple Sclerosis (MS), Rheumatoid Arthritis and Type 2 Diabetes was examined. These were chosen not as an exhaustive list of all chronic conditions, rather as merely a set of comparative examples being analogous to glaucoma: whilst they are not directly life-threatening, they all lead to disability which is likely to have a detrimental impact on QoL, typically worsening with time due to the progressive and irreversible nature of the disease [18-23]. From the example conditions assessed, those with the highest percentage of QoL studies for the last 10 years were for PD (1.6%; n = 284 out of total of 17296 studies for PD), MS (1.4% n = 260 of 18458) and AMD (1.1%; n = 60 of 5355). Hearing loss and rheumatoid arthritis had 0.7% (n = 44 of 6320) and 0.9% (n = 130 of 14412) respectively. Aside from Type 2 Diabetes (0.5%; n = 146 of 31505), glaucoma had the lowest percentage of QoL themed studies over the 10 year period (0.6%; n = 42 of 6966). Perhaps by 2009 the frequency of QOL studies in glaucoma was more similar to other chronic conditions but this represents only one year of data. Thus, the results of this comparison indicate that research in QoL in glaucoma has indeed been more neglected on the whole compared to some other example chronic conditions (Figure 2). In certain cases this is perhaps not all that surprising; for instance since AMD primarily affects central vision, its effects will be more immediately obvious as a patient attempts to carry out visually demanding tasks. Moreover, several factors may contribute to the finding that some other example conditions chosen for this study, such as PD (1.6% of total studies were QoL) and MS (1.4%) also appear to have more activity in this type of research. For example, some medical specialties, like rheumatology, have a long established history of assessment of QoL and policies to promote the use of QoL research introduced by funding bodies and professional organisations [24,25]. Moreover, as a general observation, the amount of attention paid to the impact of a disease on QoL may also be linked to how 'visible' the disability is to both the patient and those around them. This would be true of PD where the 'visible' symptoms also impact on the way the condition is perceived to the further detriment of QoL of the sufferer [26]. On the contrary, glaucoma patients themselves are often unaware of the full extent of their visual loss and their symptoms are certainly less 'noticeable' compared to other conditions [27]. Likewise, individuals with Type 2 Diabetes (which yielded the lowest number of QoL studies as a proportion of all studies in this sample) tend to be asymptomatic [28] and is described as an 'invisible' disability [26]. The impact that diabetes and glaucoma has on the individual's daily life is consequently harder for an outside observer to understand. It follows that more research should perhaps be conducted on the effect of these 'invisible' disabilities on QoL, a notion that is being addressed in the literature [29-32]. Moreover, in glaucoma, perhaps more attention should be paid to the research findings that have already challenged the belief that glaucoma is simply an insidious process in which the symptoms do not appear until the end stage of the disease [33,34].

## Methods used in QoL assessment in glaucoma

### Self-report questionnaires

Of the glaucoma-themed papers emerging from the literature search, 82% (n = 42) primarily involved the use of questionnaires to investigate the impact of glaucoma on the individual's daily functioning. The majority of these studies issued one or more established questionnaires with three main types; those measuring global health (13 studies included at least one of this type of questionnaire), those tailored towards vision-based outcomes (n = 22), and those developed especially for individuals with glaucoma (n = 9). A further four studies were dedicated to the development or validation of glaucoma-specific QoL questionnaires. An alternative method is the utility or time-trade-off approach, which typically requires judgements such as whether the individual would be willing to sacrifice a certain number of years of their life in 'exchange' for good vision: four papers included such a method. In addition, 11 studies included a novel questionnaire specifically developed for that study. Five studies involved the use of interview as opposed to written questionnaires. References for all studies identified in the glaucoma QoL search can be found in "Additional file 1".

Self-report questionnaires provide a valuable insight into the patient's personal experiences and how they perceive the impact of the disease on their life outside the clinic. It has been demonstrated that this important information is not acquired within the time-constraints of clinical appointments [35]. Individuals with glaucoma have been shown to display a poor understanding of their condition [36], and by asking them to comment on their outcomes the patient is forced to consider the impact of the disease on their own life. A collation of the data would also be useful for devising more patient-centric educational strategies. On the other hand, the usefulness of questionnaires as a QoL tool may be compromised by their subjective nature, as responses are of course difficult to verify, and are subject to bias which can manifest in a number of ways: for example, the knowledge that something is wrong with their vision has been shown to cause the patient to exaggerate symptoms and difficulties [20] or give responses they believe presents them in a socially acceptable or more favourable light [37]. Moreover, people with different expectations will self report in questionnaires that they have a different QoL even when they have exactly the same clinical condition [38]. A patient's view of their QoL may also change over time due to the manner in which they psychologically adapt to their condition [39,40]. Nevertheless, since such questionnaires provide the only feasible means of ascertaining the ways in which the patient themselves feels glaucoma has impacted on their QoL, the fact that so few studies have been dedicated to the development and validation of glaucoma-specific questionnaires is surprising.

### Performance-based studies

An alternative approach for understanding visual disability in glaucoma is to measure the patient's actual performance in surrogates of tasks they would encounter every day. The results from the survey indicated fewer studies (18% of studies [n = 9]) have taken this approach in glaucoma research. In these studies, the tasks investigated were: mobility performance in terms of ability to navigate around an obstacle course (n = 2); driving using simulations or direct on-road performance (n = 2); reading ability (n = 2); postural sway and balance (n = 2) and eye-hand co-ordination (n = 1).

This apparent lack of performance based research in glaucoma is surprising as, although the systematic control needed for this type of experiment probably places limits on how realistic the study can be, there is some evidence that task performance in the laboratory correlates well with how individuals carry out the tasks in their own home [41]. The performance-based method also helps eliminate the types of biases which coincide with the self-report method. Moreover, since glaucoma is typically perceived as asymptomatic until a more advanced stage, it is possible that performance-based assessments could capture difficulties in activities that the individual may not be sufficiently consciously aware to be able to report them in a questionnaire [42]. The development of objective measures could also potentially offer clinicians a valuable patient-centred method to monitor disease progression and to develop and evaluate the effects of management strategies. However, performance-based measures of visual disability are likely not a sufficient substitute for questionnaires because they don't, for example, measure 'feelings', symptoms or well being. Thus, it may be that disability should be measured using a combination of clinical, self-report and performance based measures to get an overall picture of the impact of glaucoma on the patient's life. This approach was recently taken in the Salisbury Eye Evaluation (SEE) Project [43,44] where both patient perceptions of QoL using the NEI-VFQ-25 and their performance in a number of daily tasks such as locating objects and using the telephone were examined, and related to the extent of visual loss.

## Conclusions

The importance of QoL studies in glaucoma recently appears to have been better recognised as the proportion of papers published relating to QoL as a surrogate measure of research activity in the area is increasing. Nevertheless, QoL studies in glaucoma represent only a very small fraction of the total publications in glaucoma research each year. Furthermore, there are fewer QoL papers in glaucoma compared to QoL type studies in some other disabling chronic conditions, including AMD. The majority of those QoL studies published in glaucoma research are based on self-report questionnaires; despite limitations this method is the best way of measuring the patient's perspective on their disease. Studies reliant on performance-based measures of visual disability are rarer but offer an additional method of increasing awareness of visual disability caused by glaucoma. Further research in these areas could prove invaluable for future management and rehabilitation in glaucoma. In turn this could help shift emphasis from the clinic towards the individuals facing the impact of visual loss on their daily lives.

## Competing interests

The authors declare that they have no competing interests.

## Authors' contributions

FCG participated in the design of the study, collection and analysis of the data and drafting of the manuscript. DPC was involved in the conception and design of the study and revision of the manuscript. DFGH provided guidance in terms of study design. All authors read and approved the final manuscript.

## Pre-publication history

The pre-publication history for this paper can be accessed here:

http://www.biomedcentral.com/1471-2415/11/19/prepub

## Supplementary Material

Additional file 1 **References for papers identified in glaucoma QoL search**. Click here for file
