# Supplementary material for: The direction of research into visual disability and quality of life in glaucoma
Source: BMC Ophthalmol. 2011 Aug 4;11:19. doi: 10.1186/1471-2415-11-19 (PMC3161024; doi:10.1186/1471-2415-11-19)
Supplement: Additional file 1 — References for papers identified in glaucoma QoL search. [file 1471-2415-11-19-S1.DOC]

**Appendix**

References for papers identified in the glaucoma QoL search; these have been assigned to appropriate categories as specified in the section entitled *Methods used in QoL assessment in glaucoma*. Note that some papers utilise a combination of measurement techniques and thus may appear in multiple categories.

**Self-report questionnaires**

*Studies using established questionnaire(s) measuring global health*

Chen L,Yang X,Chen R**.** [**Clinical observation of mental and personality characteristics of primary angle-closure glaucoma patients**](http://en.cnki.com.cn/Article_en/CJFDTOTAL-LCYZ200906027.htm). *Journal of Clinical Ophthalmology* 2009, **6.**

Iester M, Zingirian M: **Quality of life in patients with early, moderate and advanced glaucoma**. *Eye (Lond)* 2002, **16**(1):44-49.

Jampel HD, Frick KD, Janz NK, Wren PA, Musch DC, Rimal R, Lichter PR: **Depression and mood indicators in newly diagnosed glaucoma patients**. *Am J Ophthalmol* 2007, **144**(2):238-244.

Jampel HD, Schwartz A, Pollack I, Abrams D, Weiss H, Miller R: **Glaucoma patients' assessment of their visual function and quality of life**. *J Glaucoma* 2002, **11**(2):154-163.

Janz NK, Wren PA, Lichter PR, Musch DC, Gillespie BW, Guire KE: **Quality of life in newly diagnosed glaucoma patients : The Collaborative Initial Glaucoma Treatment Study**. *Ophthalmology* 2001, **108**(5):887-897.

Jayawant SS, Bhosle MJ, Anderson RT, Balkrishnan R: **Depressive symptomatology, medication persistence, and associated healthcare costs in older adults with glaucoma**. *J Glaucoma* 2007, **16**(6):513-520.

Mills RP: **Correlation of quality of life with clinical symptoms and signs at the time of glaucoma diagnosis**. *Trans Am Ophthalmol Soc* 1998, **96**:753-812.

Nah YS, Seong GJ, Kim CY: **Visual function and quality of life in Korean patients with glaucoma**. *Korean J Ophthalmol* 2002, **16**(2):70-74.

Parrish RK, 2nd, Gedde SJ, Scott IU, Feuer WJ, Schiffman JC, Mangione CM, Montenegro-Piniella A: **Visual function and quality of life among patients with glaucoma**. *Arch Ophthalmol* 1997, **115**(11):1447-1455.

Ringsdorf L, McGwin G, Jr., Owsley C: **Visual field defects and vision-specific health-related quality of life in African Americans and whites with glaucoma**. *J Glaucoma* 2006, **15**(5):414-418.

Skalicky S, Goldberg I: **Depression and quality of life in patients with glaucoma: a cross-sectional analysis using the Geriatric Depression Scale-15, assessment of function related to vision, and the Glaucoma Quality of Life-15**. *J Glaucoma* 2008, **17**(7):546-551.

Wandell PE, Lundstrom M, Brorsson B, Aberg H: **Quality of life among patients with glaucoma in Sweden**. *Acta Ophthalmol Scand* 1997, **75**(5):584-588.

Warrian KJ, Spaeth GL, Lankaranian D, Lopes JF, Steinmann WC: **The effect of personality on measures of quality of life related to vision in glaucoma patients**. *Br J Ophthalmol* 2009, **93**(3):310-315.

*Studies using established vision-based questionnaire(s)*

Bhargava JS, Patel B, Foss AJ, Avery AJ, King AJ: **Views of glaucoma patients on aspects of their treatment: an assessment of patient preference by conjoint analysis**. *Invest Ophthalmol Vis Sci* 2006, **47**(7):2885-2888.

Carrasco-Font C, Lorenzo-Martinez S, Gili-Manzanaro P, Arias-Puente A, Andres-Alba Y, Matilla-Rodriguez A, Ortigueira JA: **[Influence of visual function on quality of life in patients with glaucoma]**. *Arch Soc Esp Oftalmol* 2008, **83**(4):249-255.

Freeman EE, Munoz B, West SK, Jampel HD, Friedman DS: **Glaucoma and quality of life: the Salisbury Eye Evaluation**. *Ophthalmology* 2008, **115**(2):233-238.

Jampel HD, Frick KD, Janz NK, Wren PA, Musch DC, Rimal R, Lichter PR: **Depression and mood indicators in newly diagnosed glaucoma patients**. *Am J Ophthalmol* 2007, **144**(2):238-244.

Jampel HD, Schwartz A, Pollack I, Abrams D, Weiss H, Miller R: **Glaucoma patients' assessment of their visual function and quality of life**. *J Glaucoma* 2002, **11**(2):154-163.

Janz NK, Musch DC, Gillespie BW, Wren PA, Niziol LM: **Evaluating clinical change and visual function concerns in drivers and nondrivers with glaucoma**. *Invest Ophthalmol Vis Sci* 2009, **50**(4):1718-1725.

Janz NK, Wren PA, Lichter PR, Musch DC, Gillespie BW, Guire KE: **Quality of life in newly diagnosed glaucoma patients : The Collaborative Initial Glaucoma Treatment Study**. *Ophthalmology* 2001, **108**(5):887-897.

Labiris G, Katsanos A, Fanariotis M, Zacharaki F, Chatzoulis D, Kozobolis VP: **Vision-specific quality of life in Greek glaucoma patients**. *J Glaucoma*, **19**(1):39-43.

Magacho L, Lima FE, Nery AC, Sagawa A, Magacho B, Avila MP: **Quality of life in glaucoma patients: regression analysis and correlation with possible modifiers**. *Ophthalmic Epidemiol* 2004, **11**(4):263-270.

Maharajah KR, Tet CM, Yaacob A, Tajudin LS, Foster PJ: **Modified Bahasa Malaysia version of VF-14 questionnaire: assessing the impact of glaucoma in rural area of Malaysia**. *Clin Experiment Ophthalmol* 2008, **36**(3):222-231.

McKean-Cowdin R, Wang Y, Wu J, Azen SP, Varma R: **Impact of visual field loss on health-related quality of life in glaucoma: the Los Angeles Latino Eye Study**. *Ophthalmology* 2008, **115**(6):941-948.

Mills RP: **Correlation of quality of life with clinical symptoms and signs at the time of glaucoma diagnosis**. *Trans Am Ophthalmol Soc* 1998, **96**:753-812.

Mills RP, Janz NK, Wren PA, Guire KE: **Correlation of visual field with quality-of-life measures at diagnosis in the Collaborative Initial Glaucoma Treatment Study (CIGTS)**. *J Glaucoma* 2001, **10**(3):192-198.

Nah YS, Seong GJ, Kim CY: **Visual function and quality of life in Korean patients with glaucoma**. *Korean J Ophthalmol* 2002, **16**(2):70-74.

Parrish RK, 2nd, Gedde SJ, Scott IU, Feuer WJ, Schiffman JC, Mangione CM, Montenegro-Piniella A: **Visual function and quality of life among patients with glaucoma**. *Arch Ophthalmol* 1997, **115**(11):1447-1455.

Ringsdorf L, McGwin G, Jr., Owsley C: **Visual field defects and vision-specific health-related quality of life in African Americans and whites with glaucoma**. *J Glaucoma* 2006, **15**(5):414-418.

Rossi GC, Tinelli C, Pasinetti GM, Milano G, Bianchi PE: **Dry eye syndrome-related quality of life in glaucoma patients**. *Eur J Ophthalmol* 2009, **19**(4):572-579.

Rouland JF, Denis P, Bechetoille A, Rigeade MC, Brouquet Y, Arnould B, Baudouin C, Renard JP, Bron A, Nordmann JP *et al*: **[Creating a specific quality-of- life questionnaire in patients with glaucoma: item generation]**. *J Fr Ophtalmol* 2002, **25**(8):785-794.

Skalicky S, Goldberg I: **Depression and quality of life in patients with glaucoma: a cross-sectional analysis using the Geriatric Depression Scale-15, assessment of function related to vision, and the Glaucoma Quality of Life-15**. *J Glaucoma* 2008, **17**(7):546-551.

Skorkovska K, Cesnekova T, Skorkovska S: **[Awareness and quality of life in patients with glaucoma]**. *Cesk Slov Oftalmol* 2009, **65**(3):97-101.

Warrian KJ, Spaeth GL, Lankaranian D, Lopes JF, Steinmann WC: **The effect of personality on measures of quality of life related to vision in glaucoma patients**. *British Journal of Ophthalmology* 2009, **93**(3):310-315.

Yamagishio K, Keiji Y, Kimura T, Yamabayashi S, Katsushima H: **[Quality of life evaluation in elderly normal tension glaucoma patients using the Japanese version of VFQ-25]**. *Nippon Ganka Gakkai Zasshi* 2009, **113**(10):964-971.

*Studies using established glaucoma-specific questionnaire(s)*

Bechetoille A, Arnould B, Bron A, Baudouin C, Renard JP, Sellem E, Brouquet Y, Denis P, Nordmann JP, Rigeade MC *et al*: **Measurement of health-related quality of life with glaucoma: validation of the Glau-QoL 36-item questionnaire**. *Acta Ophthalmol* 2008, **86**(1):71-80.

Goldberg I, Clement CI, Chiang TH, Walt JG, Lee LJ, Graham S, Healey PR: **Assessing quality of life in patients with glaucoma using the Glaucoma Quality of Life-15 (GQL-15) questionnaire**. *J Glaucoma* 2009, **18**(1):6-12.

Lee BL, Gutierrez P, Gordon M, Wilson MR, Cioffi GA, Ritch R, Sherwood M, Mangione CM: **The Glaucoma Symptom Scale. A brief index of glaucoma-specific symptoms**. *Arch Ophthalmol* 1998, **116**(7):861-866.

Nelson P, Aspinall P, Papasouliotis O, Worton B, O'Brien C: **Quality of life in glaucoma and its relationship with visual function**. *J Glaucoma* 2003, **12**(2):139-150.

Perfetti S, Varotto A, Massagrandi S, Pagliani F, Bonomi L: **Glaucoma and quality of the life**. *Acta Ophthalmologica Scandinavica* 1998, **76**(S227):52-52.

Ringsdorf L, McGwin G, Jr., Owsley C: **Visual field defects and vision-specific health-related quality of life in African Americans and whites with glaucoma**. *J Glaucoma* 2006, **15**(5):414-418.

Rossi GC, Tinelli C, Pasinetti GM, Milano G, Bianchi PE: **Dry eye syndrome-related quality of life in glaucoma patients**. *Eur J Ophthalmol* 2009, **19**(4):572-579.

Skalicky S, Goldberg I: **Depression and quality of life in patients with glaucoma: a cross-sectional analysis using the Geriatric Depression Scale-15, assessment of function related to vision, and the Glaucoma Quality of Life-15**. *J Glaucoma* 2008, **17**(7):546-551.

Zanlonghi X, Arnould B, Bechetoille A, Baudouin C, Bron A, Denis P, Nordmann JP, Renard JP, Rigeade MC, Rouland JF *et al*: **[Glaucoma and quality of life]**. *J Fr Ophtalmol* 2003, **26 Spec No 2**(2):S39-44.

*Studies involving the development or validation of glaucoma-specific questionnaires*

Bechetoille A, Arnould B, Bron A, Baudouin C, Renard JP, Sellem E, Brouquet Y, Denis P, Nordmann JP, Rigeade MC *et al*: **Measurement of health-related quality of life with glaucoma: validation of the Glau-QoL 36-item questionnaire**. *Acta Ophthalmol* 2008, **86**(1):71-80.

Burr JM, Kilonzo M, Vale L, Ryan M: **Developing a preference-based Glaucoma Utility Index using a discrete choice experiment**. *Optom Vis Sci* 2007, **84**(8):797-808.

Lee BL, Gutierrez P, Gordon M, Wilson MR, Cioffi GA, Ritch R, Sherwood M, Mangione CM: **The Glaucoma Symptom Scale. A brief index of glaucoma-specific symptoms**. *Arch Ophthalmol* 1998, **116**(7):861-866.

Rouland JF, Denis P, Bechetoille A, Rigeade MC, Brouquet Y, Arnould B, Baudouin C, Renard JP, Bron A, Nordmann JP *et al*: **[Creating a specific quality-of-life questionnaire in patients with glaucoma: item generation]**. *J Fr Ophtalmol* 2002, **25**(8):785-794.

*Studies involving utility/time-trade-off approach*

Aspinall PA, Johnson ZK, Azuara-Blanco A, Montarzino A, Brice R, Vickers A: **Evaluation of quality of life and priorities of patients with glaucoma**. *Invest Ophthalmol Vis Sci* 2008, **49**(5):1907-1915.

Burr JM, Kilonzo M, Vale L, Ryan M: **Developing a Preference-Based Glaucoma Utility Index Using a Discrete Choice Experiment**. *Optom Vis Sci* 2007, **84**(8):797-808.

Gupta V, Srinivasan G, Mei SS, Gazzard G, Sihota R, Kapoor KS: Utility values among glaucoma patients: an impact on the quality of life. British Journal of Ophthalmology 2005, 89(10):1241-1244

Jampel HD, Schwartz A, Pollack I, Abrams D, Weiss H, Miller R: **Glaucoma patients' assessment of their visual function and quality of life**. *J Glaucoma* 2002, **11**(2):154-163.

*Studies including novel questionnaire developed purposely for that investigation*

Adler G, Bauer MJ, Rottunda S, Kuskowski M: **Driving habits and patterns in older men with glaucoma**. *Soc Work Health Care* 2005, **40**(3):75-87.

Fujita K, Yasuda N, Nakamoto K, Fukuda T: **[The relationship between difficulty in daily living and binocular visual field in patients with glaucoma]**. *Nippon Ganka Gakkai Zasshi* 2008, **112**(5):447-450.

Hamelin N, Blatrix C, Brion F, Mathieu C, Goemaere I, Nordmann JP: **[How patients react when glaucoma is diagnosed?]**. *J Fr Ophtalmol* 2002, **25**(8):795-798.

Haymes SA, LeBlanc RP, Nicolela MT, Chiasson LA, Chauhan BC: **Risk of Falls and Motor Vehicle Collisions in Glaucoma**. *Invest Ophthalmol Vis Sci* 2007, **48**(3):1149-1155.

Janz NK, Wren PA, Guire KE, Musch DC, Gillespie BW, Lichter PR: **Fear of blindness in the Collaborative Initial Glaucoma Treatment Study: patterns and correlates over time**. *Ophthalmology* 2007, **114**(12):2213-2220.

Nelson P, Aspinall P, O'Brien C: **Patients' perception of visual impairment in glaucoma: a pilot study**. *Br J Ophthalmol* 1999, **83**(5):546-552.

Odberg T, Jakobsen JE, Hultgren SJ, Halseide R: **The impact of glaucoma on the quality of life of patients in Norway. I. Results from a self-administered questionnaire**. *Acta Ophthalmol Scand* 2001, **79**(2):116-120.

Odberg T, Jakobsen JE, Hultgren SJ, Halseide R: **The impact of glaucoma on the quality of life of patients in Norway. II. Patient response correlated to objective data**. *Acta Ophthalmol Scand* 2001, **79**(2):121-124.

Rumiko I, Tairo K, Kunihiko F, Atsushi K, Yutaka I: **The life style of glaucoma patients.** *Japanese Journal of Clinical Ophthalmology 2000, 54(1):89-94.*

Uenishi Y, Tsumura H, Miki T, Shiraki K: **Quality of life of elderly Japanese patients with glaucoma**. *Int J Nurs Pract* 2003, **9**(1):18-25.

Viswanathan AC, McNaught AI, Poinoosawmy D, Fontana L, Crabb DP, Fitzke FW, Hitchings RA: **Severity and Stability of Glaucoma: Patient Perception Compared With Objective Measurement**. *Arch Ophthalmol* 1999, **117**(4):450-454.

*Studies involving the use of interview*

Anghel G, Anghel AC: **[Opinions about quality of life in glaucoma patients with medications in Romania]**. *Oftalmologia* 2008, **52**(3):32-35.

Bhargava JS, Patel B, Foss AJ, Avery AJ, King AJ: **Views of glaucoma patients on aspects of their treatment: an assessment of patient preference by conjoint analysis**. *Invest Ophthalmol Vis Sci* 2006, **47**(7):2885-2888.

Green J, Siddall H, Murdoch I: **Learning to live with glaucoma: a qualitative study of diagnosis and the impact of sight loss**. *Soc Sci Med* 2002, **55**(2):257-267.

Perfetti S, Varotto A, Massagrandi S, Pagliani F, Bonomi L: **Glaucoma and quality of the life**. *Acta Ophthalmologica Scandinavica* 1998, **76**(S227):52-52.

Rouland JF, Denis P, Bechetoille A, Rigeade MC, Brouquet Y, Arnould B, Baudouin C, Renard JP, Bron A, Nordmann JP *et al*: **[Creating a specific quality-of-life questionnaire in patients with glaucoma: item generation]**. *J Fr Ophtalmol* 2002, **25**(8):785-794.

***Performance based measures***

*Navigation around obstacle course*

Friedman DS, Freeman E, Munoz B, Jampel HD, West SK: **Glaucoma and mobility performance: the Salisbury Eye Evaluation Project**. *Ophthalmology* 2007, **114**(12):2232-2237.

Turano KA, Rubin GS, Quigley HA: **Mobility Performance in Glaucoma**. *Invest Ophthalmol Vis Sci* 1999, **40**(12):2803-2809.

*Driving*

Haymes SA, LeBlanc RP, Nicolela MT, Chiasson LA, Chauhan BC: **Glaucoma and**

**On-Road Driving Performance**. *Invest Ophthalmol Vis Sci* 2008, **49**(7):3035-3041.

Szlyk JP, Mahler CL, Seiple W, Edward DP, Wilensky JT: **Driving Performance of Glaucoma Patients Correlates With Peripheral Visual Field Loss**. *Journal of Glaucoma* 2005, **14**(2):145-150.

*Reading*

Fujita K, Yasuda N, Oda K, Yuzawa M: **Reading performance in patients with central visual field disturbance due to glaucoma**. *Nippon Ganka Gakkai Zasshi* 2006, **110**(11):914-918.

Ramulu PY, West SK, Munoz B, Jampel HD, Friedman DS: **Glaucoma and Reading Speed: The Salisbury Eye Evaluation Project**. *Arch Ophthalmol* 2009, **127**(1):82-87.

*Postural sway and balance*

Black AA, Wood JM, Lovie-Kitchin JE, Newman BM: **Visual impairment and postural sway among older adults with glaucoma**. *Optom Vis Sci* 2008, **85**(6):489-497.

Shabana N, Cornilleau-Peres V, Droulez J, Goh JC, Lee GS, Chew PT: **Postural stability in primary open angle glaucoma**. *Clin Experiment Ophthalmol* 2005, **33**(3):264-273

*Eye-hand coordination*

Kotecha A, O'Leary N, Melmoth D, Grant S, Crabb DP: **The Functional Consequences of Glaucoma for Eye-Hand Coordination**. *Invest Ophthalmol Vis Sci* 2009, **50**(1):203-213
